# Complete mitochondrial genome of the highly fecund *Bombyx mori* linnaeus, 1758 (Lepidoptera: Bombycidae) strain Jam 146

**DOI:** 10.1080/23802359.2021.1920860

**Published:** 2021-07-06

**Authors:** Seong-Wan Kim, Jeong Sun Park, Min Jee Kim, Kee-Young Kim, Seong-Ryul Kim, Iksoo Kim

**Affiliations:** a Department of Agricultural Biology, National Academy of Agricultural Science, Rural Development Administration, Wanju Gun, Republic of Korea; b Department of Applied Biology, College of Agriculture & Life Sciences, Chonnam National University, Gwangju, Republic of Korea; c Experiment and Analysis Division, Honam Regional Office, Animal and Plant Quarantine Agency, Gunsan, Republic of Korea

**Keywords:** Mitochondrial genome; *Bombyx mori*, Silkworm strain, Jam 146, Phylogeny

## Abstract

To meet the increasing demands of the society in the current era, new strains of the domesticated silkworm *Bombyx mori* Linnaeus, 1758 (Lepidoptera: Bombycidae) are being continuously bred. Consequently, cataloging the genetic information of pure lines is essential. The strain Jam 146, whose larvae have atypical pale, crescent-shaped body markings, is an important breeding resource due to its excellent fecundity. In this study, we sequenced the mitochondrial genome (mitogenome) of this strain using next-generation sequencing. The complete genome of this strain has a gene arrangement typical of Lepidoptera. The length of the Jam 146 mitogenome (15,661 bp) is well within the range reported in other *B. mori* strains, i.e. between 15,629 (Baiyun strain, China) and 15,676 bp (Hukpyobeom strain, South Korea). However, the total length of protein-coding genes, 3,733 codons in Jam 146 and two other silkworm strains previously reported from South Korea, is 13 codons longer than that in other *B. mori* strains. Phylogenetic analysis of 22 silkworm strains from nine countries showed that the Jam 146 strain forms a strong cluster with three other strains from China, Japan, and South Korea, suggesting that after their split from a common ancestor, the evolutionary divergence among the silkworm strains in these countries has been limited.

A wide variety of domesticated silkworm (*Bombyx mori*; Lepidoptera: Bombycidae) strains have been reported worldwide, particularly in Asia (Nagaraju 2000). Due to the increase in biomedical and cosmetic application of silk (Meinel et al. 2005; Aramwit et al. 2009; Chung et al. 2015), there is a great interest in South Korea for breeding silkworm strains that produce a high quality and quantity of the silk yarn. Larvae of strain Jam 146 have a pair of pale, crescent-shaped body markings on the second abdominal segment of the dorsal part, unlike other South Korean strains that exhibit having dark brown crescents (Kang et al. 2001). Jam 146 strain has excellent fecundity compared to other pure-line stocks. Therefore, it is used to breed a new strain Chunsujam, which yields a large quantity of silk (Kang et al. 2001). Thus, availability of mitochondrial genome (mitogenome) data for Jam 146 strain would assist bio-identification during its breeding process, along with providing important genetic information. In this study, we sequenced the complete mitogenome of the Jam 146 strain and analyzed its mitogenomic characteristics and phylogenetic position in the context of *B. mori* strains present worldwide.

One Jam 146 larva was collected from the annual culture samples of the government institute in South Korea and subsequently deposited at Chonnam National University, South Korea, under accession number CNU8264 (Iksoo Kim, ikkim81@chonnam.ac.kr). Mitogenome sequences were constructed by *de novo* assembly using a GenBank-registered silkworm mitogenome (NC_002355; unpublished) after whole genome sequencing was performed on the NextSeq-500 platform (Illumina, San Diego, CA, USA). No Sanger-based gap filling was conducted due to certainty of the final genome sequence. The genomic sequences were compared with those of 21 strains from nine countries (Figure 1), including two from South Korea (Li et al. 2010; Zhang et al. 2016; Kim et al. 2019a, 2019b).

**Figure 1.**
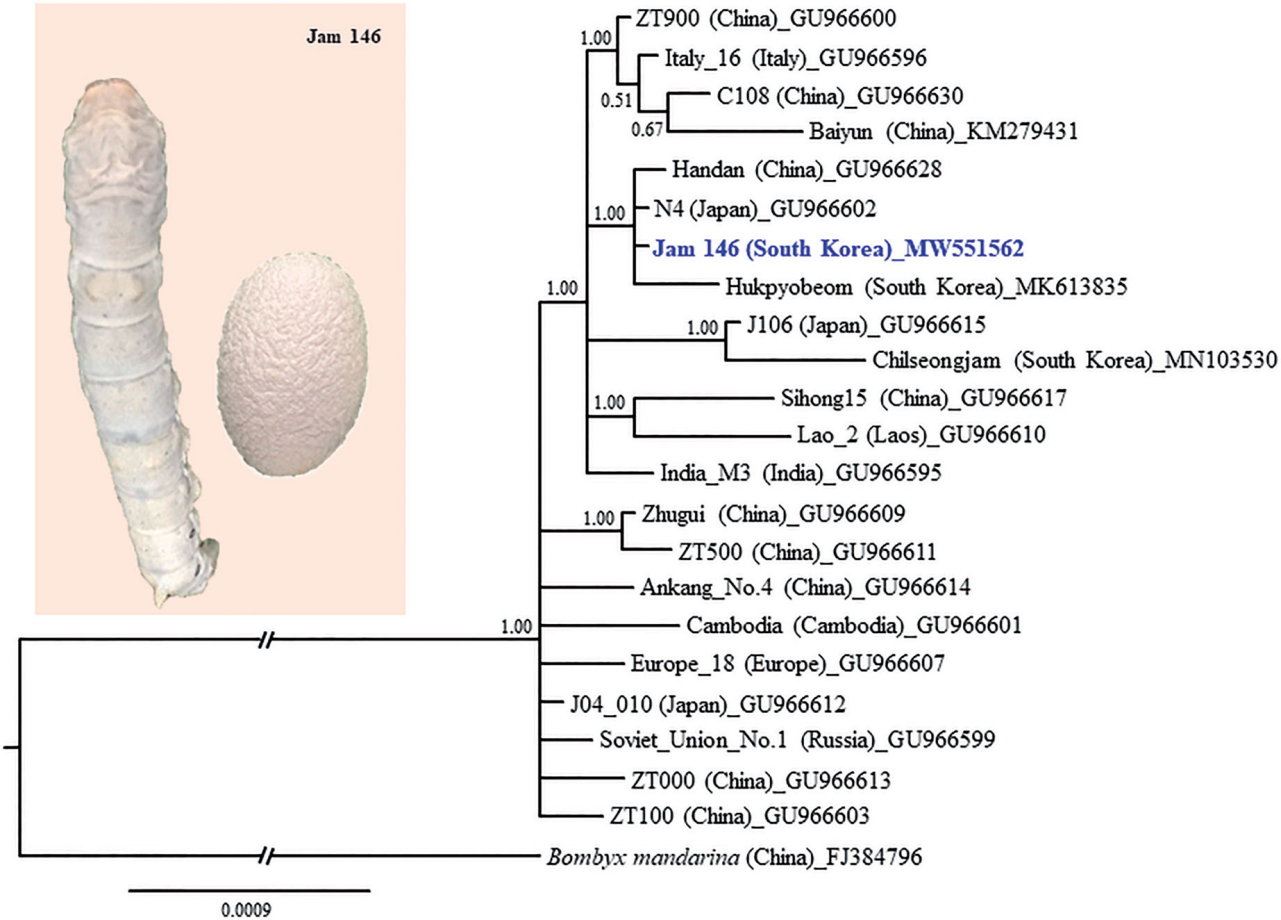
Phylogenetic tree of *Bombyx mori*. The Bayesian inference (BI) method, based on the concatenated sequences of 13 protein-coding genes and two ribosomal RNAs, was used for phylogenetic analysis. The GenBank accession numbers are provided next to strain name. The numbers at the node specify Bayesian posterior probabilities. The scale bar indicates the number of substitutions per site. The wild silkworm, *Bombyx mandarina* (Hu et al. 2010), was designated as the outgroup based on previous phylogenetic results (Wang et al. 2019). Publication information is as follows: Baiyun, Zhang et al. (2016); Jam 146, This study; Hukpyobeom, Kim et al. (2019a); Chilseongjam, Kim et al. (2019b); and the remaining strains, Li et al. (2010). The branch length was truncated to approximately one-third of the original length due to limited space.

The complete mitogenome of Jam 146 (15,661 bp) is composed of typical gene sets and a major non-coding A + T-rich region (GenBank acc. no. MW551562); the gene arrangement is identical to that of ditrysian Lepidoptera species (Park et al. 2016). The genome has typical start and stop codons, including the CGA start codon for COI, which is found almost universally in Lepidoptera (Kim et al. 2010; Park et al. 2016). In strain Jam 146, the A/T content was 81.37% % in whole genome, 79.60% in PCGs, 81.78**%** in tRNAs, 84.40% in lrRNA, 85.53% in srRNA, and 95.55% in the A + T-rich region. The AT content in other strains, used for phylogenetic analysis ranged from 81.32 (the Baiyun strain, Zhang et al. 2016) to 81.40% (Kim et al. 2019a). Divergence in whole genome sequence among 22 strains ranged from 0.012% (C108 vs. ZT900; Li et al. 2010) to 0.438% (Heukpyobeom vs. Baiyun; Zhang et al. 2016; Kim et al. 2019a). Compared with the genome of 19 other *B. mori* strains reported by Li et al. (2010) and Zhang et al. (2016), genome size of Jam 146 is 5 bp longer on average; however, it is 32 bp longer than that of the BaiyuN strain. Furthermore, genome size of Jam 146 is 15 bp shorter and 1 bp longer than that of Hukpyobeom and Chilseongjam (the two previously reported South Korea strains), respectively (Kim et al. 2019a, 2019b). Importantly, these three South Korean strains, including Jam 146, have a total of 3,733 codons for protein coding genes (PCGs), whereas strains from other countries have 3,720 codons (Li et al. 2010; Zhang et al. 2016).

Using the Bayesian inference (BI) method of phylogenetic analysis with concatenated sequences of 13 PCGs and two rRNA genes (Miller et al. 2010; Stamatakis 2014), we showed that Jam 146 forms a strong monophyletic group (Bayesian posterior probabilities = 1) with three other strains from China, Japan, and South Korea. This suggests that these four strains are phylogenetically the most similar, regardless of where they are currently preserved. This indicates the existence of little evolutionary divergence among silkworm strains of these countries, despite silkworms being bred into diverse strains following domestication of the ancestral species. Similarly, other proposed groups with high nodal support also indicate similar grouping patterns, further reinforcing low evolutionary divergence among strains. We hope that the mitogenome sequence of strain Jam 146 reported here will be useful as a strain-diagnostic marker and for further in-depth genetic studies of domesticated silkworm strains worldwide.

## Data Availability

The genome sequence data supporting the findings of this study are openly available in GenBank of NCBI at https://www.ncbi.nlm.nih.gov/nuccore/MW551562.1
